# *Phyllanthus emblica* aqueous extract retards hepatic steatosis and fibrosis in NAFLD mice in association with the reshaping of intestinal microecology

**DOI:** 10.3389/fphar.2022.893561

**Published:** 2022-07-26

**Authors:** Xiaomin Luo, Boyu Zhang, Yehua Pan, Jian Gu, Rui Tan, Puyang Gong

**Affiliations:** ^1^ College of Pharmacy, Southwest Minzu University, Chengdu, China; ^2^ College of Life Science and Engineering, Southwest Jiaotong University, Chengdu, China

**Keywords:** nonalcoholic fatty liver disease, *Phyllanthus emblica*, gut microbiota, fecal metabolites, hepatic fibrosis

## Abstract

Accumulating evidence suggests that dysregulation of the intestinal flora potentially contributes to the occurrence and development of nonalcoholic fatty liver disease (NAFLD). *Phyllanthus emblica* (PE), an edible and medicinal natural resource, exerts excellent effects on ameliorating NAFLD, but the potential mechanism remains unclear. In the present study, a mouse NAFLD model was established by administering a choline-deficient, L-amino acid-defined, high-fat diet (CDAHFD). The protective effects of the aqueous extract of PE (AEPE) on the gut microbiota and fecal metabolites in NAFLD mice were detected by performing 16S rRNA gene sequencing and untargeted metabolomics. The administration of middle- and high-dose AEPE decreased the levels of ALT, AST, LDL-C, TG, and Hyp and increased HDL-C levels in CDAHFD-fed mice. Hematoxylin–eosin (H&E), Oil Red O, and Masson’s trichrome staining indicated that AEPE treatment attenuated hepatic steatosis and fibrotic lesions. Moreover, the disordered intestinal microflora was remodeled by AEPE, including decreases in the abundance of *Peptostreptococcaceae*, *Faecalibaculum*, and *Romboutsia*. The untargeted metabolomics analysis showed that AEPE restored the disturbed glutathione metabolism, tryptophan metabolism, taurine and hypotaurine metabolism, and primary bile acid biosynthesis of the gut bacterial community in NAFLD mice, which strongly correlated with hepatic steatosis and fibrosis. Collectively, AEPE potentially ameliorates NAFLD induced by a CDAHFD through a mechanism associated with its modulatory effects on the gut microbiota and microbial metabolism.

## 1 Introduction

Nonalcoholic fatty liver disease (NAFLD) is characterized by the excessive accumulation of lipids in hepatocytes of individuals without alcohol abuse or other specific liver damage factors (Ren et al., 2021). The global incidence of NAFLD is approximately 25%, and NAFLD has become the most frequent cause of chronic liver disease worldwide with the increasing prevalence of obesity and metabolic syndrome (Vernon et al., 2011; Wong and Chan, 2021). NAFLD, which is characterized by a spectrum of hepatic pathology, ranges from simple hepatic steatosis to nonalcoholic steatohepatitis (NASH), which further progresses to fibrosis, cirrhosis, and eventually liver carcinoma (Song et al., 2020). At the NASH stage, hepatic steatosis coexists with inflammation, causing progressive fibrosis to develop into long-term complications (Li et al., 2022a). However, currently, approved therapeutic agents to restrain and reverse the progression of NAFLD are still unavailable (Schwabe et al., 2020; Parlati et al., 2021).

Based on accumulating evidence, the gut microbiome–liver axis plays an important role in NAFLD, especially in progression toward a more advanced disease stage (Tilg et al., 2021). Alterations in the structure and abundance of the gut microbiota might influence the metabolism of lipids, carbohydrates, and amino acids, which contribute to the development of NAFLD (Chen et al., 2021; Ren et al., 2021). The changes of the intestinal microbiota could promote the incidence and progression of NAFLD (Diehl and Day, 2017; Fang et al., 2018; Kolodziejczyk et al., 2019; Tilg et al., 2020). A study on microbial transplantation showed that sterile mice receiving gut microbiota from high-fat diet (HFD)-induced NAFLD mice increased liver fat accumulation and reduced short-chain fatty acid production, and the changes were more pronounced than in simple diet-induced NAFLD (Porras et al., 2019). Likewise, germ-free mice colonized with cecum contents from HFD-induced NAFLD mice exhibited hepatic steatosis and increased NAFLD susceptibility (Le Roy et al., 2013). Furthermore, extensive studies have demonstrated that disruption of metabolites mediated by intestinal flora, such as bile acids, tryptophan, and branched-chain amino acids, can induce the development or worsening of NAFLD (Ni et al., 2020). These studies highlight that modulation of gut microecology may be a new strategy to prevent or treat NAFLD.

*Phyllanthus emblica* L. (PE) is an edible and medicinal homologous plant belonging to the family *Euphorbiaceae*, which is widely distributed in subtropical and tropical areas of China and India (Variya et al., 2016). It possesses the properties of cooling blood, eliminating food, and suppressing cough and is used to treat heat in blood, indigestion, and hepatobiliary disease (Saini et al., 2022). Extensive pharmacodynamic studies have reported that PE extracts exert remarkable effects on ameliorating liver diseases, such as viral hepatitis, alcoholic hepatitis, NAFLD, and hepatocellular carcinoma (Yin et al., 2022). Among these extracts, the aqueous extract of PE (AEPE) has been shown to ameliorate hepatic steatosis and inflammation in a mouse model of NAFLD induced by a methionine–choline-deficient (MCD) high-fat diet (Huang et al., 2017; Tung et al., 2018). Meanwhile, our previous study showed that AEPE attenuates liver fibrosis caused by carbon tetrachloride *in vivo* (Yin et al., 2021). However, researchers have not clearly determined whether AEPE ameliorates fibrosis in subjects with NAFLD, and the mechanism by which it treats NAFLD must be further investigated.

In the current study, we first confirmed the protective effects of varying doses of AEPE on mice with CDAHFD-induced NAFLD by measuring biochemical markers and performing a histopathological examination. Then, 16S rRNA gene sequencing was performed to detect changes in the structural composition of the gut microbiota caused by AEPE treatment, and ultra-performance liquid chromatography with mass spectrometry (UPLC-MS) was applied to characterize metabolites in feces. Additionally, a correlation analysis was conducted to reveal the relationship between the altered microbiome and microbial metabolites induced by AEPE. The present study provides a novel mechanistic perspective of the role of PE in preventing the deterioration of NAFLD and facilitating the development of medicinal and healthcare applications of PE.

## 2 Materials and methods

### 2.1 Materials

Silymarin was purchased from Madaus (Cologne, Germany). The chemical reference standards, gallic acid, corilagin, and ellagic acid were purchased from Sichuan Weikeqi Biological Technology Co., Ltd. (Chengdu, China). Commercial diagnostic kits of alanine aminotransferase (ALT), aspartate aminotransferase (AST), triglyceride (TG), high-density lipoprotein cholesterol (HDL-C), low-density lipoprotein cholesterol (LDL-C), and hydroxyproline (Hyp) were obtained from Jiancheng Bioengineering Institute (Nanjing, China). Magnetic Soil and Stool DNA Kit (DP712) was obtained from Tiangen Biotech Co., Ltd. (Beijing, China). Phusion^®^ High-Fidelity PCR Master Mix was purchased from New England Biolabs LTD. (Beijing, China). GeneJET Gel Extraction Kit and Qubit^®^ 2.0 Fluorometer were purchased from Thermo Fisher Scientific. TruSeq DNA PCR-Free Library Preparation Kit was purchased from Illumina Co., Ltd.

Methanol, formic acid, and ammonium acetate were purchased from Sigma-Aldrich Chemical Co. (St. Louis, MO, United States). LC–MS grade water was purchased from Merck KGaA (Darmstadt, Germany). The dried *Phyllanthus emblica* L. (PE) fruits were obtained from the Lotus Pond herbal medicine market (Chengdu, China) and were authenticated by Prof. Jian Gu from the College of Pharmacy, Southwest Minzu University. The voucher specimen (No. 20201212) has been deposited at the herbarium of the College of Pharmacy, Southwest Minzu University.

### 2.2 Preparation and analysis of the chemical profile of AEPE

AEPE was prepared as described in our previous study (Yin et al., 2021). Briefly, 200 g of dried PE power was added to 2,000 ml of distilled water and extracted on a rotary shaker (150 rpm) at 37°C for 24 h. The extract solutions were filtrated and concentrated to 200 ml. The HPLC analysis was performed using an Agilent 1260 HPLC system equipped with a Kromasil 100-5-C18 column (4.6 × 250 mm, 5 μm). The mobile phase consisted of water (A) and acetonitrile containing 0.1% formic acid (B) as follows: 3% B (0–6 min), 3%–4% B (6–15 min), 4%–14% B (15–20 min), 14% B (20–50 min), and 14%–40% B (50–80 min). The UV detection wavelength was set at 254 nm. The flow rate was 0.6 ml/min, and the injection volume was 5 μl. The column temperature was maintained at 37°C. The constituents were quantified using external standards based on the analytical curves of gallic acid (y = 13006x + 97.108, *r* ^2^ = 0.9996, 0.25–4 mg ml^−1^), corilagin (y = 12259x − 721.79, *r* ^2^ = 0.9995, 0.16–3.5 mg ml^−1^), and ellagic acid (y = 13488x − 161.95, *r* ^2^ = 0.9995, 0.16–4 mg ml^−1^).

### 2.3 Experimental animals and treatment

Five-week-old male C57BL/6J mice (18–22 g, specific pathogen free) were purchased from GemPharmatech Co., Ltd. (Jiangsu, China) and maintained at a controlled temperature (22.0 ± 2°C) and relative humidity (50%–60%) on a 12-h light/12-h dark cycle with free access to food and drink. All animal procedures were approved by the Animal Ethics Committee of Southwest Minzu University.

**FIGURE 1:**
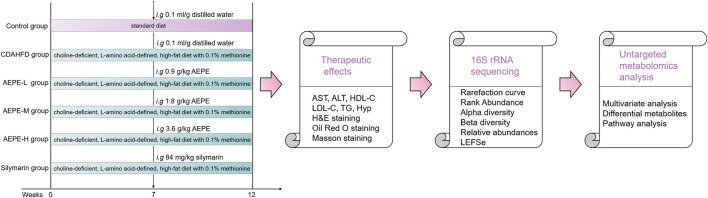
Overview of the experimental design for all groups.

After acclimation and feeding for 1 week, the mice were randomly assigned to two groups: the control group (control), which received a standard diet (11% kcal fat, TP36225MCS, Trophic Animal Feed High-tech Co., Ltd., Jiangsu, China, *n* = 10), and the CDAHFD groups, which were fed a choline-deficient, L-amino acid-defined, high-fat diet with 0.1% methionine (65% kcal fat, TP36225MCD, Trophic Animal Feed High-tech Co., Ltd, Jiangsu, China, *n* = 50). After 6 weeks, mice in CDAHFD groups were randomly divided into the following five groups (*n* = 10 mice per group) according to the treatment and fed for a period of 6 weeks. The groups included the CDAHFD group, AEPE low-dose (0.9 g of crude drug/kg) treatment group (AEPE-L), AEPE middle-dose (1.8 g of crude drug/kg) treatment group (AEPE-M), AEPE high-dose (3.6 g of crude drug/kg) treatment group (AEPE-H), and 84 mg/kg silymarin treatment group (Silymarin) (Figure 1).

### 2.4 Sample collection

Mouse feces were collected daily at the same time in the twelfth week, and fecal samples from each mouse were immediately collected in 2.0-ml cryogenic vials, immersed in liquid nitrogen, and stored at −80°C until further analysis. After the last feeding, all mice were fasted for 12 h, weighed, and then completely anesthetized with 1.5% isoflurane. Blood samples were obtained from the retro-orbital plexus. The samples were incubated at 25°C for 1 h and then centrifuged at 862 *g* for 15 min to separate the serum, which was stored at −20°C. A small piece of fresh liver was removed and immersed in a tissue fixative solution (4% paraformaldehyde solution) for subsequent staining and assessment. The remainder of the liver tissue was stored at −80°C until further analysis.

### 2.5 Analysis of serum and liver biochemical parameters

Serum AST and ALT levels were determined with appropriate kits according to the manufacturer’s instructions. The contents of TG, HDL-C, LDL-C, and Hyp in the liver tissue homogenates were measured using commercially available kits.

### 2.6 Histological analysis

The liver tissue samples were fixed with 4% paraformaldehyde, processed, and embedded in paraffin for hematoxylin and eosin (H&E) staining. Slices were stained with Masson’s trichrome to observe the progression of hepatic fibrosis. Frozen sections of the liver were stained with Oil Red O to visualize lipid accumulation within the hepatocytes. Images of pathological sections were captured using an Olympus BX53 microscope.

### 2.7 Gut microbiota analysis

#### 2.7.1 Extraction of fecal genomic DNA for 16S rRNA sequencing

The total genomic DNA was extracted from fecal samples in the control, CDAHFD, and AEPE-M groups (six samples per group) using a Magnetic Soil and Stool DNA Kit according to the manufacturer’s instructions. The V3-V4 hypervariable region of the bacterial 16S rRNA gene was amplified with the primers 341F (5′-CCTAYGGGRBGCASCAG-3′) and 806R (5′-GGACTACNNGGGTATCTAAT-3′). The polymerase chain reaction (PCR) was performed with 30 and 15 μl of Phusion^®^ High Fidelity PCR Master Mix; 0.2 μM forward and reverse primers; and approximately 10 ng of template DNA. The thermal cycle involved an initial denaturation at 98°C for 1 min, denaturation at 98°C for 10 s, annealing at 50°C for 30 s, extension at 72°C for 30 s, and a final extension at 72°C for 5 min. An equal volume of loading buffer was mixed with the PCR product and detected after electrophoresis on a 2% agarose gel. A sample with a clear main band of 400–450 bp was selected for a subsequent experiment. PCR products were blended in a ratio of equal densities. The hybrid PCR products were then purified using the GeneJET Gel Extraction Kit.

#### 2.7.2 Bioinformatics analysis

A sequencing library was generated, and an index code was added using the Illumina TruSeq DNA PCR-Free Library Preparation Kit according to the manufacturer’s instructions. Library quality was assessed using a Qubit^®^ 2.0 fluorometer and an Agilent Bioanalyzer 2100 system. Ultimately, the library was sequenced with the Illumina NovaSeq platform to generate a 250-bp paired read sequence. Quality filtering of the paired-end raw reads was performed under specific filtering conditions, and paired-end clean reads were merged. 16S rRNA gene sequences were analyzed using the Quantitative Insights Into Microbial Ecology (QIIME) software (version 1.9.1). The operational taxonomic unit (OTU) cluster sequence analysis was performed using pick_de_novo_otus.py; sequences with greater than or equal to 97% similarity were assigned to the equivalent OTUs. Then, species annotation was performed on the OTU sequence and SILVA_138 database. The alpha diversity and beta diversity indices of the samples were calculated immediately afterward using the QIIME software and displayed using the R software (version 2.15.3).

### 2.8 UHPLC-MS/MS analysis of fecal metabolites

#### 2.8.1 Fecal sample preparation

Fecal samples (0.1 g) were ground in liquid nitrogen, and the homogenate was resuspended by vortexing in precooled 80% methanol. The specimens were placed on ice, incubated for 5 min, and centrifuged at 15,000 ×*g* for 20 min at 4°C. After centrifugation, a portion of the supernatant was diluted with LC-MS grade water to a final concentration containing 53% methanol. The specimens were then transferred to a new microcentrifuge tube and centrifuged at 15,000 ×*g* for 20 min at 4°C. Afterward, the supernatant was placed in an autosampler vial for further analysis.

#### 2.8.2 UHPLC-MS/MS analysis

The UHPLC-MS/MS analysis was performed using a Vanquish ultra-high-performance liquid chromatograph (UHPLC) in combination with an Orbitrap Q Exactive™ HF-X mass spectrometer. The supernatant was separated on a Hypersil GOLD column (100 × 2.1 mm, 1.9 µm) with a 17-min linear gradient and a flow rate of 0.2 ml per min. The eluents for the positive polarity mode were eluent A (0.1% formic acid in water) and eluent B (methanol). The eluates for the negatively polarized mode were eluate A (5 mm ammonium acetate, hydrogen potential of 9.0) and eluate B (methanol). The procedure for gradient elution with the solvent was as follows: 2% B, 0–1.5 min; 2%–85% B, 1.5–3 min; 100% B, 3–10 min; 100%–2% B, 10–10.1 min; and 2% B, 10.1–12 min. A Q Exactive™ HF-X spectrometer was operated in positive and negative ion mode with a voltage of 3.5 kV, the capillary temperature of 320°C, a sheath gas flow rate of 35 psi, an aux gas flow rate of 10 L per min, a S-lens RF class level of 60, and an auxiliary gas heater temperature of 350°C. The full-scan range was m/z 100–1500.

#### 2.8.3 Data processing and analysis of metabolites

The original UHPLC-MS/MS data files were manipulated using Compound Discoverer 3.1 (CD 3.1) software (Thermo Fisher) for peak alignment, peak picking, and quantification of the individual metabolites. Then, the peak intensity was normalized to the total spectral intensity. The molecular formula based on additive ions, molecular ion peaks, and fragment ions was predicted from the normalized data. Then, the mzCloud (https://www.mzcloud.org/), mzVault, and MassList databases were used to obtain accurate qualitative and relative quantitative results. Normalized data were imported into the SIMCA-P software (version 14.1; SIMCA-P). Principal component analysis (PCA), partial least squares discriminant analysis (PLS-DA), and orthogonal partial least squares discriminant analysis (OPLS-DA) were conducted to obtain clustering information and salient variables. The Kyoto Encyclopedia of Genes and Genomes (KEGG) database (https://www.genome.jp/kegg/pathway.html), Human Metabolome Database (HMDB) (https://hmdb.ca/metabolities), and LIPID MAPS database (http://www.lipidmaps.org/) were used to annotate these metabolites. MetaboAnalyst (https://www.metaboanalyst.ca/) (version 5.0) was used to identify metabolic pathways.

### 2.9 Statistical analysis

All data are presented as the means ± S.E.M. Graphical representations of the results were performed using GraphPad Prism 8.3.0 software. The significance level of differences between two groups was analyzed using Student’s unpaired t-test, while the data from multiple groups were statistically analyzed using one-way analysis of variance (ANOVA) followed by Dunnett’s multiple comparisons post hoc test. A *p*-value < 0.05 was regarded as statistically significant.

## 3 Results

### 3.1 Quantitative analysis of representative components of AEPE

**FIGURE 2:**
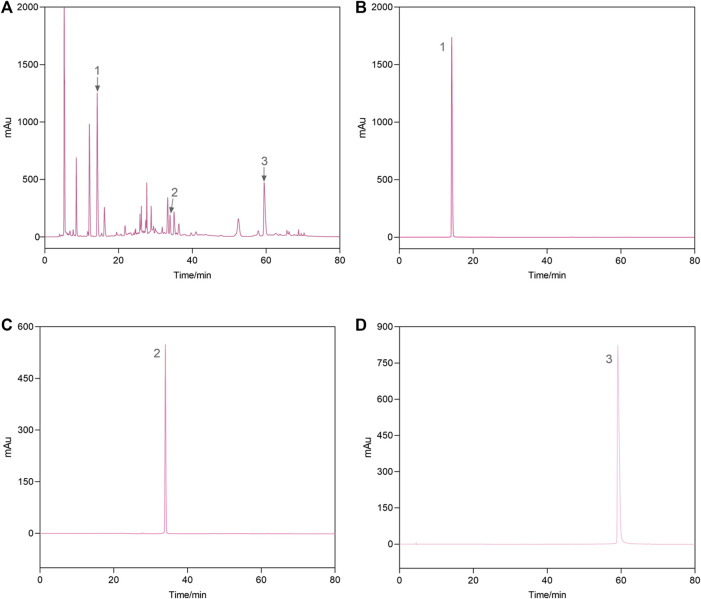
Representative high-performance liquid chromatograms of the AEPE sample **(A)** and standard compounds **(B–D)**. 1, gallic acid; 2, corilagin; 3, ellagic acid.

As shown in Figure 2, the HPLC chromatogram indicated that AEPE contained gallic acid, corilagin, and ellagic acid, with retention time peaks at 14.203, 33.938, and 59.462 min, respectively. The gallic acid, corilagin, and ellagic acid contents in PE were 14.6, 2.8, and 9.5 mg/g, respectively.

### 3.2 AEPE alleviated hepatic steatosis in mice treated with CDAHFD

**FIGURE 3:**
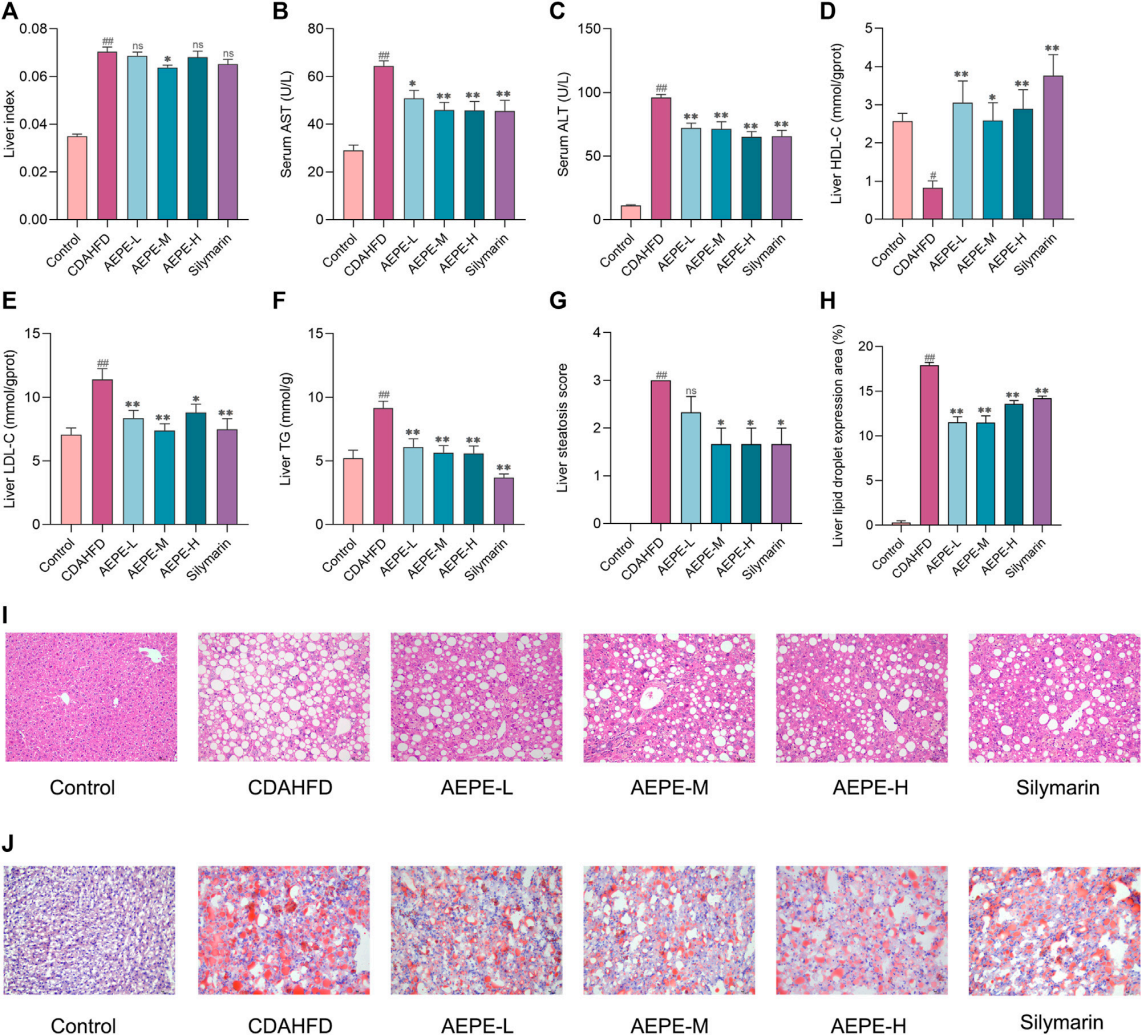
AEPE treatment attenuated hepatic steatosis in CDAHFD-fed mice. **(A)** AEPE-M treatment decreased the liver index in CDAHFD mice. **(B)** AEPE treatment decreased the level of AST in CDAHFD mice. **(C)** AEPE treatment decreased the level of ALT in CDAHFD mice. **(D)** AEPE treatment increased the level of HDL-C in CDAHFD mice. **(E)** AEPE treatment decreased the level of LDL-C in CDAHFD mice. **(F)** AEPE treatment decreased the level of TG in CDAHFD mice. **(G,I)** H&E staining showed that AEPE treatment significantly improved the pathological changes in the liver (×200). **(H,J)** Oil Red O staining showed that AEPE treatment significantly improved the accumulation of red lipid droplets in the liver (×200). Control, CDAHFD, AEPE-L, AEPE-M, AEPE-H, and silymarin (*n* = 10 per group) groups. Data were presented as the mean ± SEM. ^#^ *p* < 0.05, ^##^ *p* < 0.01 as compared to the control group; *p < 0.05, **p < 0.01 as compared to the CDAHFD group.

As illustrated in Figure 3A, a medium dose of AEPE (1.8 g of crude drug/kg) significantly reduced the elevated liver index of NAFLD mice (*p* < 0.05). Additionally, compared with the control diet, the CDAHFD triggered not only significantly abnormal serum AST and ALT levels but also abnormal LDL-C, TG, and HDL-C concentrations in liver tissue. However, AEPE (0.9, 1.8, and 3.6 g of crude drug/kg) and silymarin (84 mg/kg) treatments reversed these changes (Figures 3B–F). The results obtained here are similar to those obtained by Huang et al. (2017), who found that the water extract of PE significantly decreased the levels of ALT, AST and LDL-C in rats with HFD-induced NAFLD. Hence, the results further confirmed that AEPE improved liver function and lipid metabolism in NAFLD mice.

H&E staining and Oil Red O staining were performed to further verify the degree of hepatic steatosis. As shown in Figures 3G,I, the liver histology of mice in the CDAHFD group revealed apparent hepatocyte swelling and necrosis, balloon-like changes, and slight inflammatory cell infiltration. Compared with the CDAHFD group, the AEPE groups displayed substantial amelioration of these changes. Oil Red O staining (Figures 3H,J) showed the accumulation of numerous red lipid droplets in the CDAHFD group, which was significantly improved by AEPE and silymarin treatments. Hence, AEPE prevented NAFLD development in CDAHFD-fed mice, especially the medium dose of AEPE.

### 3.3 AEPE attenuates hepatic fibrosis progression in NAFLD mice

**FIGURE 4:**
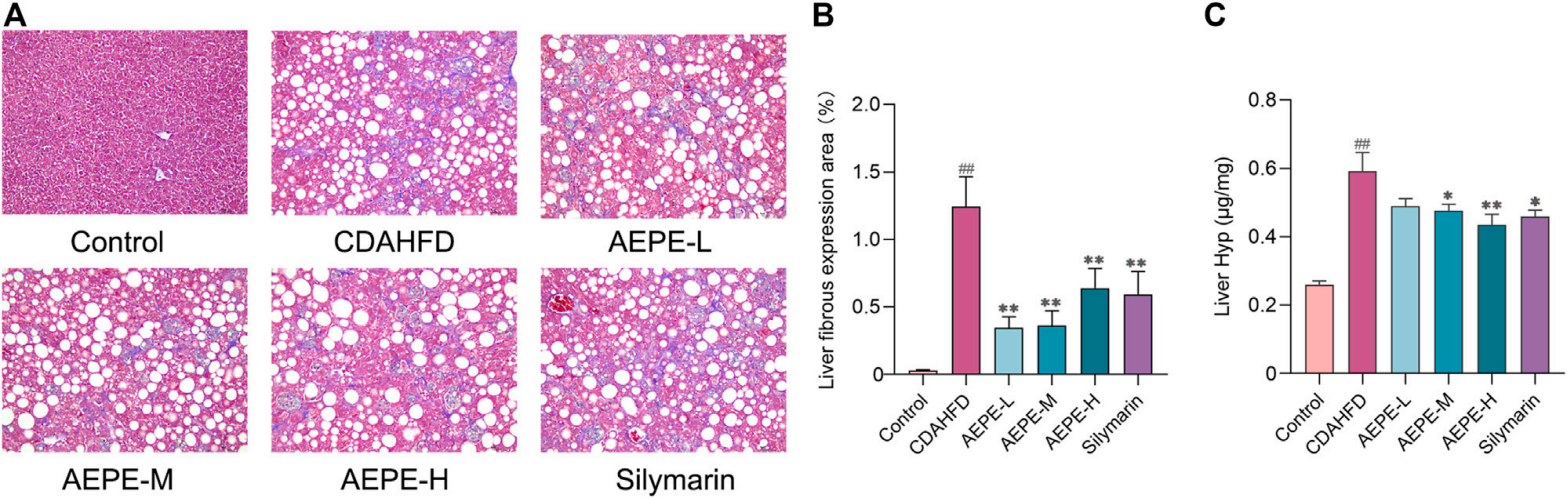
AEPE treatment attenuated hepatic fibrosis in CDAHFD-fed mice. **(A,B)** Masson’s staining showed that AEPE treatment significantly decreased blue collagen fibers in the liver (×200). **(C)** AEPE treatment decreased the level of Hyp in CDAHFD mice. Control, CDAHFD, AEPE-L, AEPE-M, AEPE-H, and silymarin (*n* = 10 per group) groups. Data are presented as the mean ± SEM. ^##^ *p* < 0.01 as compared to the control group; *p < 0.05, **p < 0.01 as compared to the CDAHFD group.

Liver fibrosis is an important pathological marker for the deterioration of NAFLD; hence, the protective effects of AEPE were further evaluated using Masson’s trichrome staining and measurements of Hyp levels. As shown in Figures 4A,B, no obvious change in collagen expression was observed in the control group, but a large amount of collagen was expressed and deposited in the liver tissue of CDAHFD mice. The AEPE intervention noticeably decreased the number of collagen fibers. Moreover, the Hyp content in the CDAHFD group increased significantly (*p* < 0.01, Figure 4C) compared to that in the control group and was significantly reduced (*p* < 0.05) following the consumption of medium and high doses of AEPE and silymarin. Taken together, these results showed that the AEPE-M treatment better prevented the progression of NAFLD.

### 3.4 AEPE-M regulates the composition of the gut microbiota that is altered by CDAHFD

**FIGURE 5:**
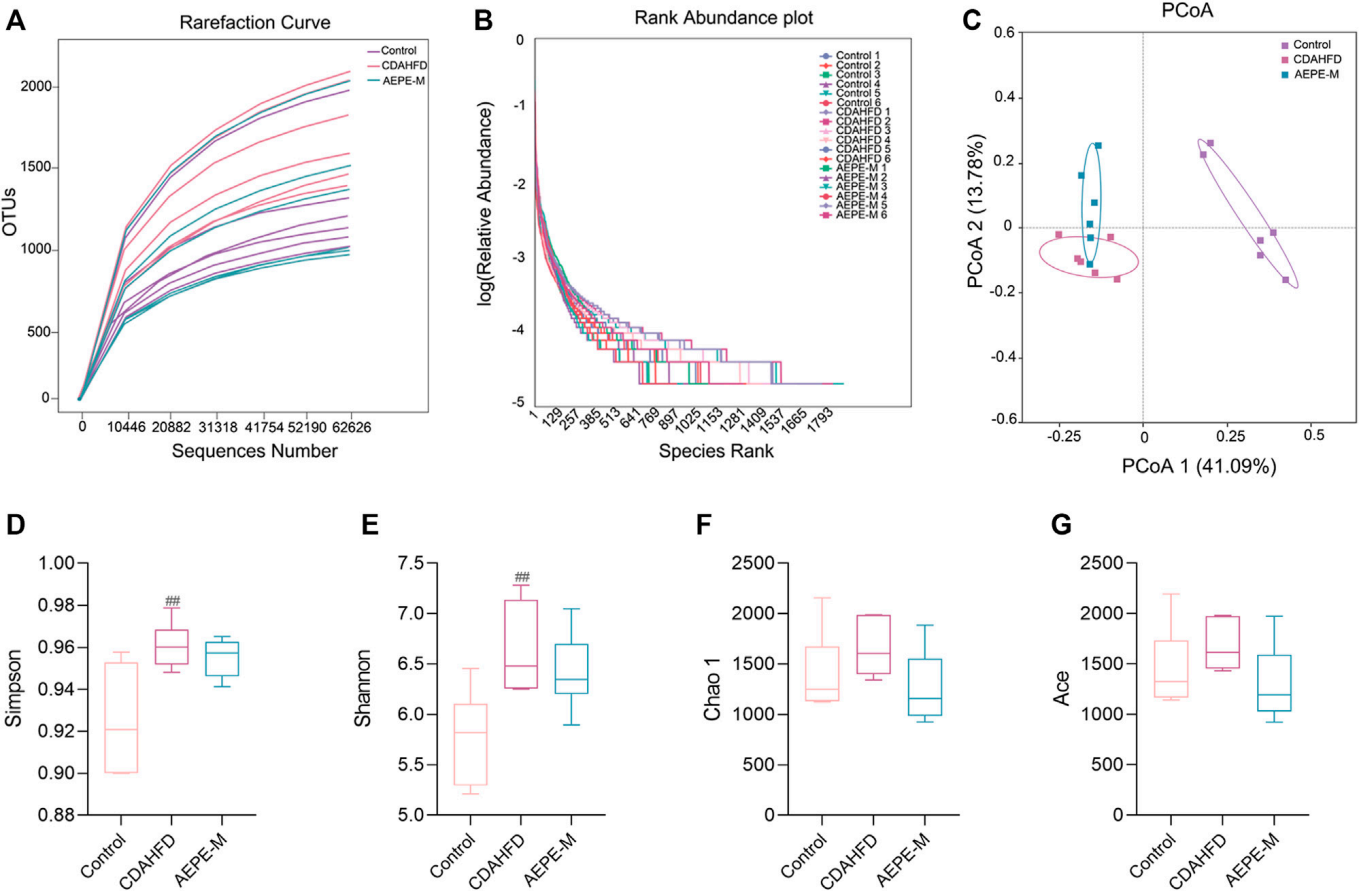
AEPE treatment affected the diversity in CDAHFD mice. **(A)** Rarefaction curve flattened out indicated that the sequencing depth has basically covered all the species in the sample. **(B)** Rank abundance indicated that the libraries were large enough to cover most of the bacterial diversity in each sample. **(C)** PCoA indicated more similar beta diversity between the AEPE-M and control groups than that between the CDAHFD and control groups. **(D,E)** Simpson’s and Shannon’s indexes were higher in the CDAHFD group than in the control group. **(F,G)** There were no significant differences in the Chao 1 index and the Ace index in each group. Control, CDAHFD, and AEPE-M (*n* = 6 per group) groups. Data are presented as the mean ± SEM. ^##^ *p* < 0.01 as compared to the control group.

A rarefaction curve has been used to determine whether the current sequencing depth of each sample adequately reflects the microbial diversity of the sample. As shown in Figure 5A, the rarefaction curve flattened out, and we considered that the sequencing depth basically covered all the species in the sample. The rank abundance showed that the libraries were sufficiently large to cover most of the bacterial diversity in each sample (Figure 5B). Based on the richness and alpha diversity analyses, Simpson’s and Shannon’s indices were significantly higher in the CDAHFD group than in the control group (*p* < 0.01; Figures 5D,E), whereas this change was moderated by the AEPE-M treatment. We performed a similarity analysis to test whether the between-group differences were greater than the within-group differences and to show that the groups were meaningful. According to the current experimental results (Table 1), by observing the R-value and *p*-value between the groups, the difference between the groups was greater than the difference within the group.

**TABLE 1:** Analysis of differences between ANOSIM groups.

Group	*R*-value	*P*-value
Control vs. AEPE-M	0.9556	0.002
CDAHFD vs. AEPE-M	0.6389	0.004
CDAHFD vs. control	0.9889	0.004

The *R*-value was found to be between −1 and 1, when *R* > 0, indicating that the between-group differences were significant, and *R* < 0 indicated that the within-group differences were greater than between-group differences. The reliability of the statistical analysis was expressed as *p*-value, and *p* < 0.05 indicated that the difference was statistically significant.

Principal coordinates analysis (PCoA) was used to evaluate the differences and similarities in the gut microbiota components of mice in the control group, CDAHFD group, and AEPE-M group (Figure 5C). As shown in the results of PCoA, the samples of the control group were significantly separated from those of the other two groups. As expected, the samples obtained after the AEPE-M intervention tended to be close to the control group. Therefore, AEPE-M and CDAHFD altered the overall composition of the gut microbiota.

**FIGURE 6:**
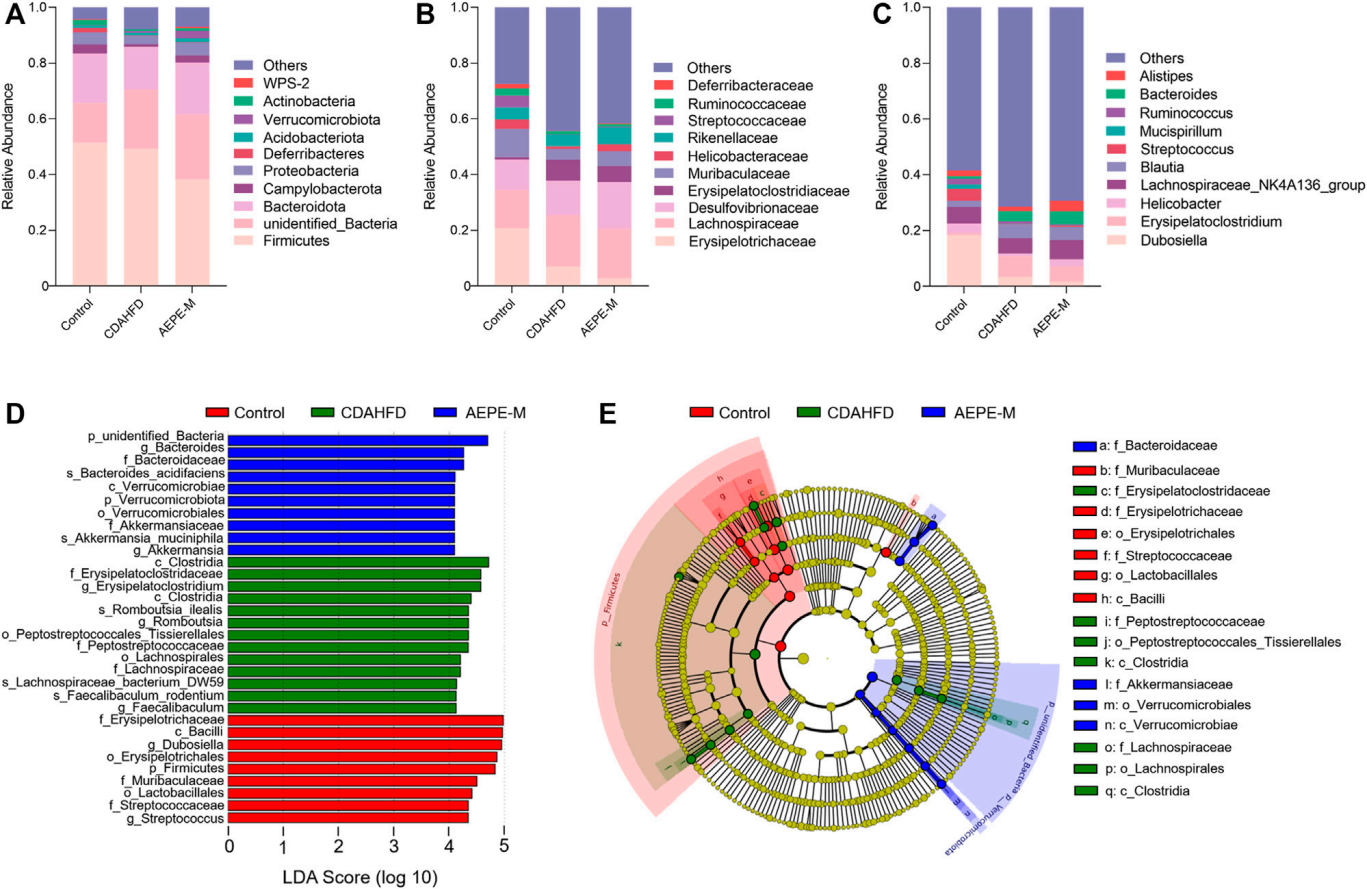
AEPE treatment affected the abundance of gut microbiota in CDAHFD mice. **(A)** At the phylum level, the top 10 relative abundances in each group. **(B)** At the family level, the top 10 relative abundances in each group. **(C)** At the genus level, the top 10 relative abundances in each group. **(D,E)** LEFSe showed significant differences in gut microbiota among groups. Control, CDAHFD, and AEPE-M (*n* = 6 per group) groups.

We assessed the bacteria with the top 10 relative abundance at the phylum (Figure 6A), family (Figure 6B), and genus (Figure 6C) levels to clarify which bacteria accounted for the differences in the intestinal microbiota structure among the different groups. At the phylum level, the relative abundance of *Firmicutes* and *Bacteroidetes* accounted for a greater proportion of the total. The ratio of *Firmicutes* to *Bacteroidetes* (F/B) in mice fed the CDAHFD was obviously higher than that in the control mice. After AEPE-M treatment, this change was reversed and reduced to levels close to the ratio in control mice. We used linear discriminant analysis effect size (LEFSe) to further identify the flora that plays an important role in the gut microbiota and showed significant differences between groups. The statistical results of the LEFSe include two parts, the linear discriminant analysis (LDA) value distribution histogram (Figure 6D) and an evolutionary branching diagram (Figure 6E). As shown in the figure, nine strains of bacteria were significantly enriched in the control group, including *Erysipelotrichaceae*, *Bacilli*, *Dubosiella*, *Erysipelotrichales*, *Firmicutes*, *Muribaculaceae*, *Lactobacillales*, *Streptococcaceae*, and *Streptococcus*. Thirteen strains of bacteria were significantly enriched in the CDAHFD group, and the relative abundance of *Clostridia*, *Erysipelatoclostridiaceae*, *Romboutsia_ilealis*, *Peptostreptococcales_Tissierellales*, and *Peptostreptococcaceae* was relatively high. A previous study reported that *Peptostreptococcaceae* was significantly elevated in NAFLD mice induced by HFD (Soares et al., 2021), which is consistent with our findings. Ten strains of bacteria that were significantly enriched in the AEPE-M group were as follows: *unidentified_Bacteria*, *Bacteroides*, *Bacteroidaceae*, *Bacteroides_acidifaciens, Verrucomicrobiae*, *Verrucomicrobiota*, *Verrucomicrobiales*, *Akkermansiaceae*, *Akkermansia_muciniphila*, and *Akkermansia*. Collectively, these findings suggest that AEPE-M supplementation regulated the composition of the intestinal flora in CDAHFD-fed mice.

**FIGURE 7:**
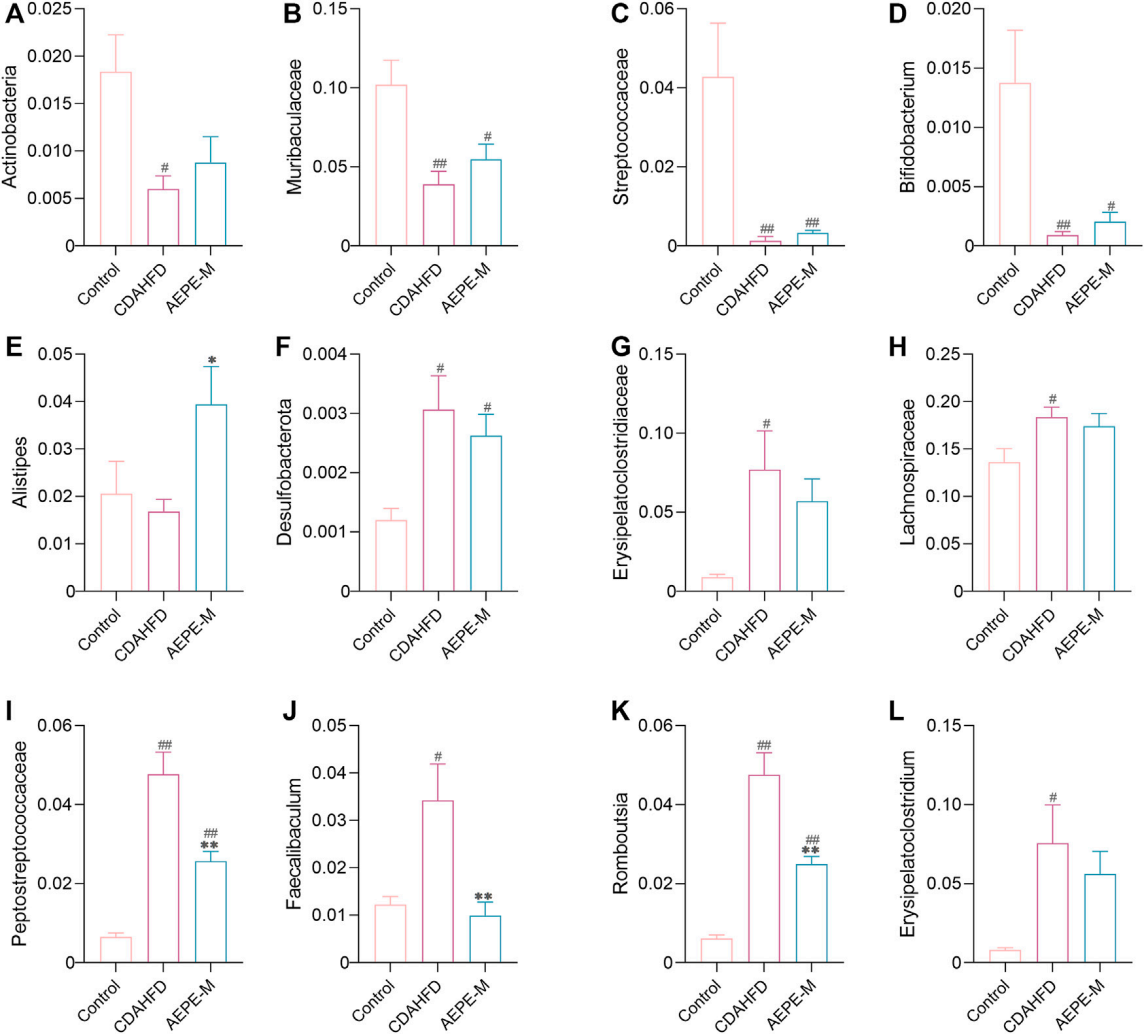
Relative abundance of mice at phylum, family, and genus levels. Compared with the control group, the relative abundance of *Actinobacteria* **(A)**, *Muribaculaceae* **(B)**, *Streptococcaceae* **(C)**, *Bifidobacterium* **(D)**, and *Alistipes* **(E)** obviously decreased in the CDAHFD group; *Desulfobacterota* **(F)**, *Erysipelatoclostridiaceae* **(G)**, *Lachnospiraceae* **(H)**, *Peptostreptococcaceae* **(I)**, *Faecalibaculum* **(J)**, *Romboutsia* **(K)**, and *Erysipelatoclostridium* **(L)** obviously increased in the CDAHFD group. AEPE treatment could effectively improve this change. Control, CDAHFD, and AEPE-M (*n* = 6 per group) groups. Data are presented as the mean ± SEM. ^#^ *p* < 0.05, ^##^ *p* < 0.01 as compared to the control group; *p < 0.05, **p < 0.01 as compared to the CDAHFD group.

Specifically, as presented in Figure 7, at the phylum level, the relative abundance of *Actinobacteria* in the control and AEPE-M groups was 1.839 ± 0.3898% and 0.8804 ± 0.2726%, respectively, which were higher than that in the CDAHFD group (0.5991 ± 0.1401%). The relative abundance of *Desulfobacterota* in the control and AEPE-M groups was 0.2626 ± 0.03626% and 0.1200 ± 0.01966%, respectively, which were slightly lower than that in the CDAHFD group (0.3068 ± 0.05706%). At the family level, the relative abundance of *Peptostreptococcaceae* in the control group was 0.6545 ± 0.09701%, which was significantly lower than that in the CDAHFD group (4.772 ± 0.5602%) (*p* < 0.01), and this change was significantly reversed by AEPE-M (2.573 ± 0.2426%) (*p* < 0.01). In addition, the relative abundance of *Muribaculaceae* and *Streptococcaceae* in the control group was 10.20 ± 1.554% and 4.280 ± 1.359%, respectively, which was significantly higher than that in the CDAHFD group (3.890 ± 0.8296% and 0.1316 ± 0.1075%, respectively) (*p* < 0.01) and AEPE-M group (5.486 ± 0.9541% and 0.3287 ± 0.06166%, respectively) (*p* < 0.05 and *p* < 0.01, respectively). At the genus level, the CDAHFD group presented significantly increased levels of *Faecalibaculum* and *Romboutsia* (3.426 ± 0.7685% and 4.757 ± 0.5592, respectively) (*p* < 0.05 and *p* < 0.01, respectively) compared with the control group (1.222 ± 0.1721% and 0.6125 ± 0.08919%, respectively), and the levels were significantly decreased when animals were treated with AEPE-M (0.9926 ± 0.2854% and 2.490 ± 0.2024%, respectively) (*p* < 0.01).

### 3.5 AEPE-M repaired metabolic disorders induced by CDAHFD

**FIGURE 8:**
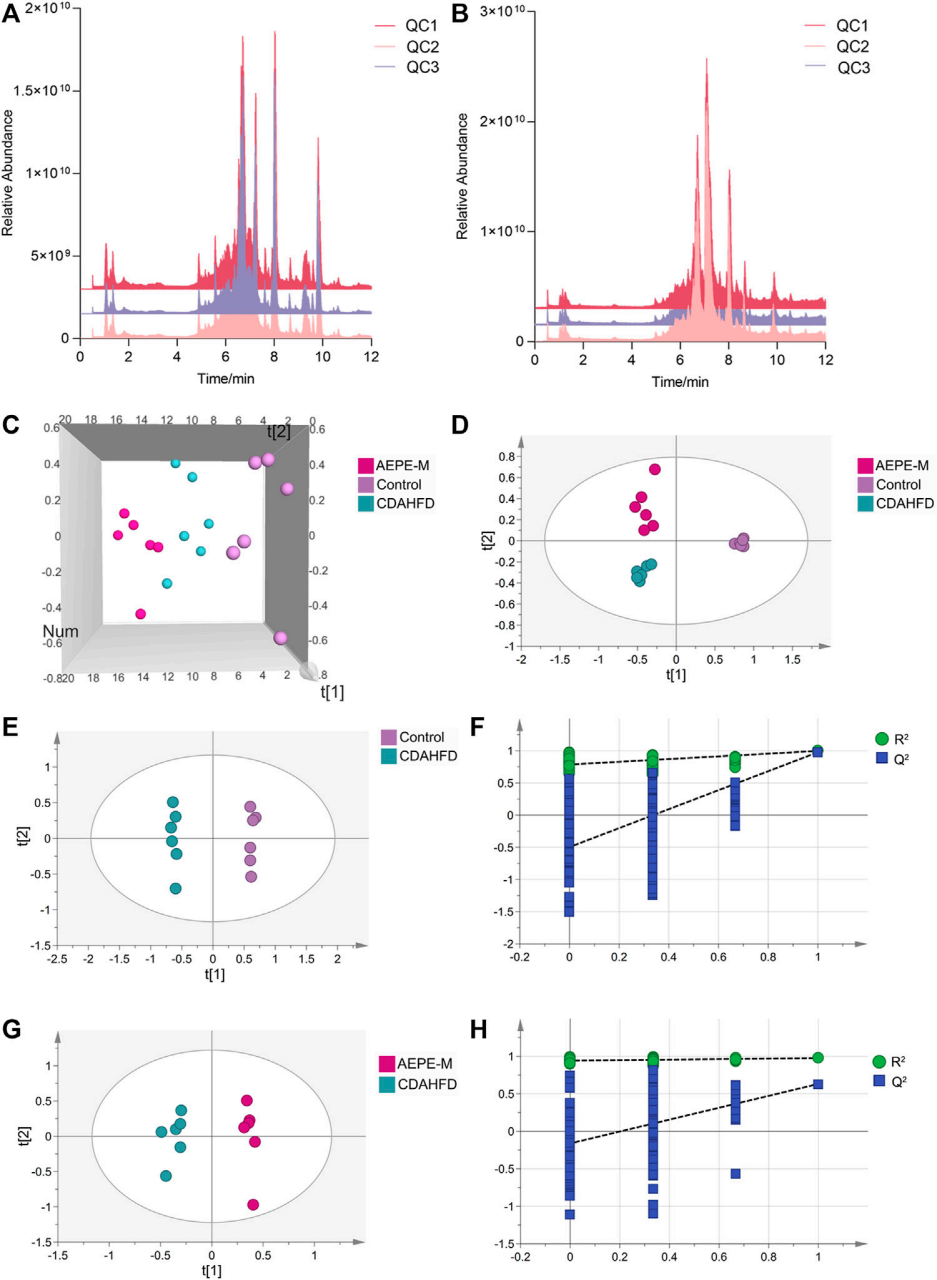
AEPE treatment modulated the fecal metabolites in CDAHFD mice. Positive **(A)** and negative **(B)** ions showed a high overlap between the TIC of different QC samples. **(C)** Score plots of 3D PCA between the control, CDAHFD, and AEPE-M groups. **(D)** Score plots of PLS-DA between the control, CDAHFD, and AEPE-M groups. **(E,F)** Score plots of OPLS-DA between the control and the CDAHFD groups and the corresponding coefficient of loading plots. **(G,H)** Score plots of OPLS-DA between the AEPE-M and CDAHFD groups and the corresponding coefficient of loading plots. Control, CDAHFD, and AEPE-M (*n* = 6 per group) groups.

UPLC–MS/MS was used to analyze the metabolites in fecal samples and systematically confirm that AEPE-M retards the progression of NAFLD by reshaping the intestinal microecology. As metabolite groups are susceptible to external factors and change rapidly, data quality control (QC) is necessary to obtain stable and accurate results for different metabolites. The total ion current (TIC) of QC samples analyzed using mass spectrometry in positive (Figure 8A) and negative (Figure 8B) ion modes showed a high overlap between the TIC of different QC samples, indicating that the mass spectrometer had better signal stability for the same sample at different times. The metabolites obtained in positive and negative ion modes were combined for further principal component analysis (PCA). As shown in the PCA 3D diagram (Figure 8C), there was a clear separation between the three groups. In addition, partial least squares discriminant analysis (PLS-DA) was performed to distinguish the differences in metabolites between the control group, CDAHFD group, and AEPE-M group, and the plot of PLS-DA scores indicated that these three groups were obviously distinguished (Figure 8D).

Subsequently, we used orthogonal partial least squares discriminant analysis (OPLS-DA) to further observe the metabolic changes between the control and CDAHFD groups, and between the CDAHFD and AEPE-M groups. The OPLS-DA models showed significant differences in metabolomic data between the control group and the CDAHFD group (Figure 8E), as well as between the CDAHFD group and the AEPE-M group (Figure 8G). After randomization to 200 replacement tests, the OPLS-DA model had *R* ^2^ and *Q* ^2^ values of 0.786 and −0.497 for the comparison of the control and CDAHFD groups, respectively (Figure 8F). The OPLS-DA model had *R* ^2^ and *Q* ^2^ values of 0.941 and −0.162 for the comparison of the CDAHFD and AEPE-M groups, respectively (Figure 8H). These results revealed that the OPLS-DA models were reliable and robust.

Metabolites with a VIP > 1, *p* < 0.05, FC > 1.2, or FC < 0.833 between the control and CDAHFD groups and the CDAHFD and AEPE-M groups were selected as differential metabolites. In positive ion mode, 407 differential metabolites were identified between the control and CDAHFD groups, 76 between the CDAHFD and AEPE-M groups, and 32 metabolites in common. In negative ion mode, 231 differential metabolites were identified between the control and CDAHFD groups, 70 between the CDAHFD and AEPE-M groups, and 16 metabolites in common. Then, these 48 differential metabolites were identified by searching a database. After removing the metabolites of nonhuman origin and drugs, 14 common differential metabolites were finally identified. Detailed information on the metabolites is shown in Table 2. The contents of glucose 1-phosphate, N-lactoyl-phenylalanine, 3-indoleacrylic acid, and glutathione disulfide decreased significantly after mice were fed the CDAHFD and increased significantly after AEPE-M treatment. The contents of the metabolites 5-hydroxytryptamine, L-ornithine, prostaglandin H2, methylmalonic acid, indoleacrylic acid, taurine, deoxycytidine, taurochenodeoxycholic acid, 3-hydroxy-3-methylbutanoic acid, and 2-(3,4-dimethoxyphenyl) ethanamine increased significantly after mice were fed the CDAHFD and decreased significantly after AEPE-M treatment.

**TABLE 2:** Differential metabolites in fecal after AEPE-M treatment.

No.	Rt (min)	m/z	Formula	Metabolite	MS/MS	VIP		FC		Trend	
						C vs. M	A vs. M	C vs. M	A vs. M	C vs. M	A vs. M
1	1.11	133.0972	C_5_H_12_N_2_O_2_	L-Ornithine	132.99745, 119.01838, 114.98771, 101.00803, 87.99341, 86.99271, 70.06506, 69.98296, 69.15391, and 68.98217	1.13	1.68	0.29	0.44	↓^##^	↓^*^
2	1.22	124.0075	C_2_H_7_NO_3_S	Taurine	127.03908, 126.09113, 126.06609, 126.05488, 109.02833, 96.04423, 84.0444, 81.03347, 80.04948, and 69.03353	1.50	1.68	0.80	0.21	↓^##^	↓^*^
3	1.29	261.0372	C_6_H_13_O_9_P	Glucose 1-phosphate	262.25247, 212.50664, 145.04935, 127.03897, 122.79235, 98.98416, 97.02851, 89.64538, 85.02845, and 72.50112	1.07	1.67	5.48	4.42	↑^##^	↑^*^
4	1.43	226.0839	C_9_H_13_N_3_O_4_	Deoxycytidine	229.00197, 228.17061, 228.08607, 210.07626, 166.0502, 157.09702, 112.05047, 84.08077, 72.0809, and 70.06501	1.14	1.40	0.52	0.66	↓^#^	↓^*^
5	2.56	117.0196	C_4_H_6_O_4_	Methylmalonic acid	117.92876, 117.01919, 116.92859, 100.9259, 99.92582, 99.00894, 80.46878, 73.0295, and 66.29874	1.21	1.80	0.40	0.49	↓^##^	↓^*^
6	4.71	119.0705	C_5_H_10_O_3_	3-Hydroxy-3-methylbutanoic acid	129.1019, 104.96317, 102.94769, 100.51136, 91.05414, 86.99245, 86.95286, 72.93713, 68.98221, and 56.94242	1.45	2.03	0.11	0.37	↓^##^	↓^*^
7	4.80	177.1023	C_10_H_12_N_2_O	5-Hydroxytryptamine	-	1.01	2.23	0.23	0.42	↓^##^	↓^*^
8	4.97	238.1076	C_12_H_15_NO_4_	N-Lactoyl-phenylalanine	238.14291, 238.1068, 238.07872, 224.06406, 124.03922, 106.02879, 96.04425, 78.03376, 73.04677, and 69.06993	1.60	1.41	3.43	1.71	↑^##^	↑^*^
9	5.67	182.1180	C_10_H_15_NO_2_	2-(3,4-Dimethoxyphenyl) ethanamine	182.10657, 182.08101, 165.07375, 164.07042, 137.0786, 136.07561, 122.0599, 108.08056, 93.06973, and 91.05442	1.24	1.74	0.50	0.63	↓^##^	↓^*^
10	5.70	164.0359	C_8_H_7_NO_3_	Indoleacrylic acid	167.07295, 167.05589, 166.07224, 149.04567, 138.0547, 135.02998, 124.05039, 121.02822, 120.08071, and 74.02355	1.24	1.86	0.54	0.64	↓^##^	↓^*^
11	6.40	188.0707	C_11_H_9_NO_2_	3-Indoleacrylic acid	188.07043, 170.05988, 146.05989, 144.08067, 143.07275, 142.06517, 118.06502, 117.06979, 115.05425, and 86.09643	1.17	2.39	1.81	2.14	↑^##^	↑^*^
12	6.99	498.2893	C_26_H_45_NO_6_S	Taurochenodeoxycholic acid	501.23895, 500.37692, 500.31119, 500.24118, 454.30618, 377.17145, 156.0768, 110.07115, 95.0603, and 83.06045	1.28	1.68	0.26	0.36	↓^#^	↓^*^
13	7.37	333.2074	C_20_H_32_O_5_	Prostaglandin H2	353.24774, 335.23715, 252.18697, 211.14816, 183.1169, 157.10127, 155.0856, 143.08546, 129.06982, and 95.08549	1.46	1.56	0.37	0.59	↓^##^	↓^*^
14	7.55	657.1525	C_20_H_32_N_6_O_12_S_2_	Glutathione disulfide	—	1.62	1.44	3.58	1.71	↑^##^	↑^**^

Control, CDAHFD, AEPE-M (*n* = 6 per group) groups. ^#^ *p* < 0.05, ^##^ *p* < 0.01 as compared to the control group; *p < 0.05, **p < 0.01 as compared to the AEPE-M group; ↑, content increased; ↓, content decreased; vs., versus; C, control group; M, CDAHFD group; A, AEPE-M group.

### 3.6 Analysis of metabolic pathways of differential metabolites

**FIGURE 9:**
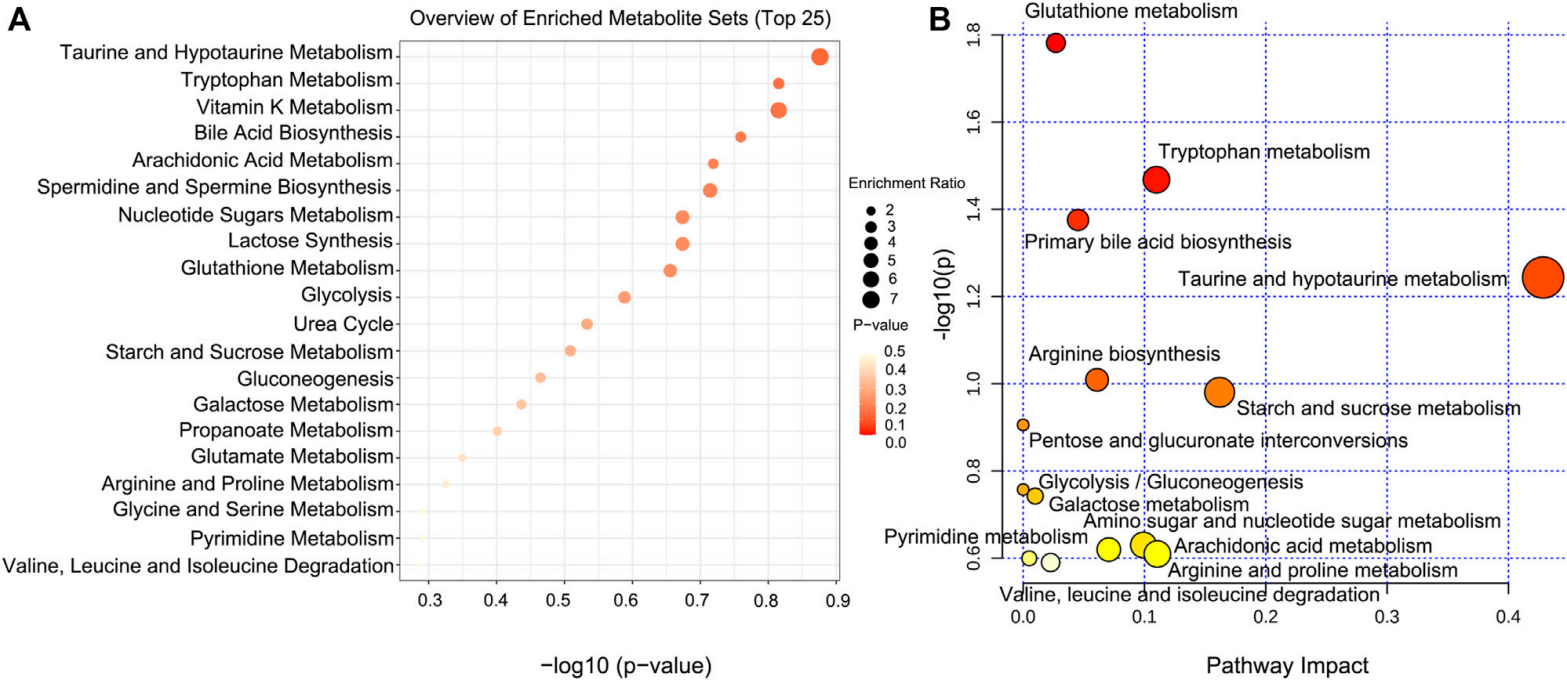
Pathway analysis of significantly altered metabolites for NAFLD. **(A)** Visual analysis of enrichment pathway of altered metabolites. **(B)** Pathway analysis of typical metabolites in response to NAFLD. Each dot represents a metabolic pathway.

According to the position of metabolites in related pathways, topology and pathway enrichment analyses were conducted to evaluate the roles of differential metabolites in biological reactions and determine the involved metabolomic pathways. All differential metabolites were imported into MetaboAnalyst for the metabolic pathway analysis. The analysis results are presented in visual form, as shown in Figures 9A,B. The results of metabolic pathway enrichment and topological analysis indicated that the treatment of CDAHFD-induced NAFLD mice was mainly related to the metabolic pathways of glutathione metabolism, tryptophan metabolism, taurine and hypotaurine metabolism, primary bile acid biosynthesis, arginine biosynthesis, starch and sucrose metabolism, pentose and glucuronate interconversions, glycolysis/gluconeogenesis, galactose metabolism, arachidonic acid metabolism, amino sugar and nucleotide sugar metabolism, arginine and proline metabolism, pyrimidine metabolism, and valine, leucine, and isoleucine degradation.

### 3.7 Correlation analysis of physiological data, fecal differential metabolites, and differential microorganisms

**FIGURE 10:**
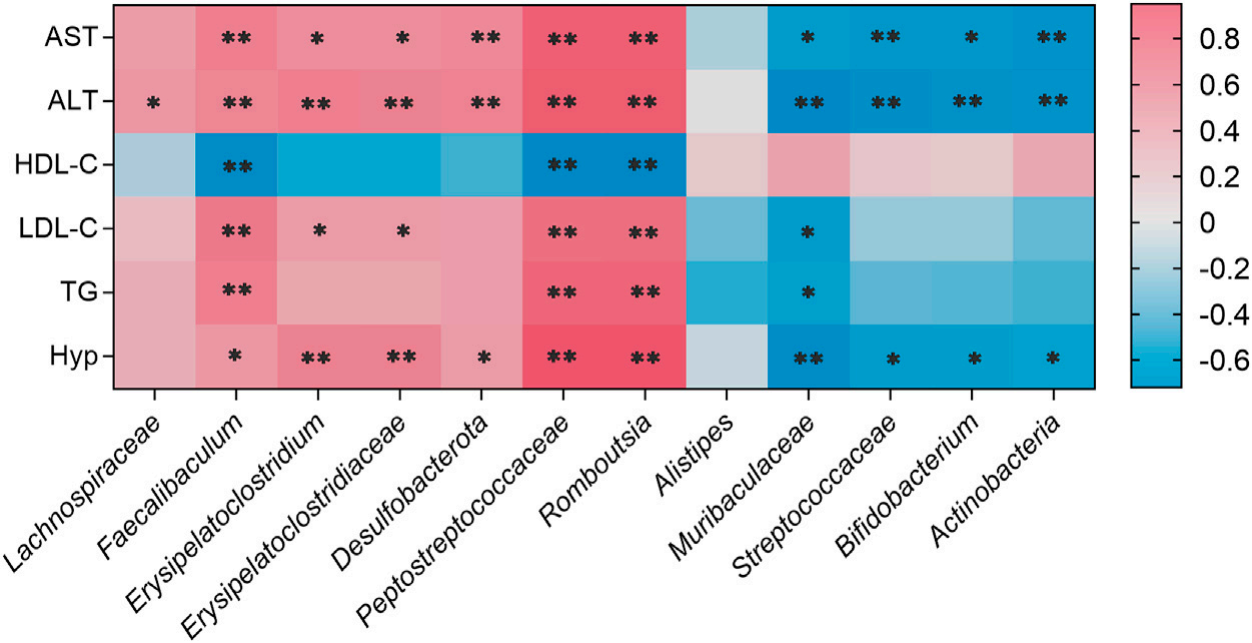
Correlations between physiological data and gut microbiota were analyzed using Spearman’s analysis (heatmap). The x-axis represents the gut microbiota with differential abundance. The y-axis represents the physiological data. The colors of grids represent the value of Spearman’s correlation analysis. Grids in red indicate positive correlations (correlation analysis value greater than 0.1), while grids in blue indicate negative correlations (correlation analysis value less than −0.1). Color coding scale indicates the correlation analysis value from heatmap; the deeper red or blue indicates higher correlation values. **p < 0.01 between physiological data and gut microbiota. *p < 0.05 between physiological data and gut microbiota.

As shown in Figure 10, *Faecalibaculum*, *Erysipelatoclostridium*, *Erysipelatoclostridiaceae*, *Desulfobacterota*, *Peptostreptococcaceae*, and *Romboutsia* showed significant positive correlations with changes in AST, ALT, LDL-C, TG, and Hyp levels in NAFLD mouse models. *Faecalibaculum*, *Peptostreptococcaceae,* and *Romboutsia* showed significant negative correlations with changes in HDL-C levels in NAFLD mouse models. *Muribaculaceae*, *Streptococcaceae*, *Bifidobacterium,* and *Actinobacteria* exhibited significant negative correlations with changes in AST, ALT, and Hyp levels in NAFLD mouse models.

**FIGURE 11:**
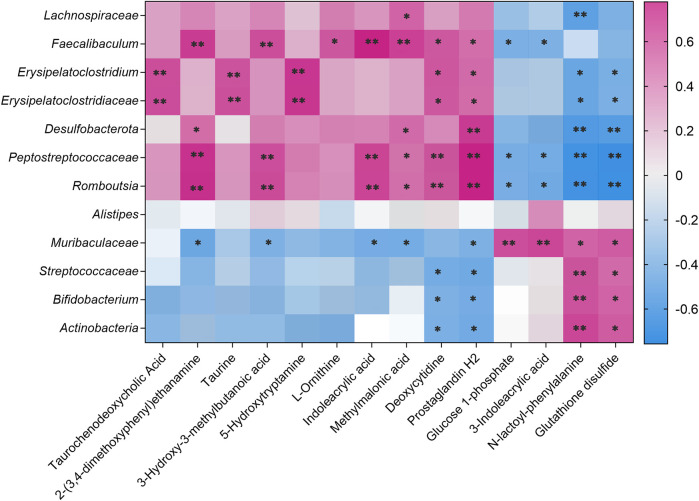
Correlation analysis of untargeted metabolomics and 16S rRNA sequencing. Correlations between untargeted metabolomics and gut microbiota were analyzed using Spearman’s analysis (heatmap). The x-axis represents the differential metabolites in the fecal. The y-axis represents the gut microbiota with differential abundance. The colors of grids represent the correlation analysis value of Spearman’s correlation analysis. Grids in purple indicate positive correlations (correlation analysis value greater than 0.1), while grids in blue indicate negative correlations (correlation analysis value less than −0.1). Color coding scale indicates the correlation analysis value from heatmap; the deeper purple or blue indicates higher correlation values. **p < 0.01 between fecal metabolites and gut microbiota. *p < 0.05 between fecal metabolites and gut microbiota.

Pearson’s correlation analysis was performed to determine the correlation between the three groups with differences in the gut microbiota and their metabolites. The result was presented in a clustered heatmap that illustrated the relative increasing (purple) or decreasing (blue) trends, as shown in Figure 11. *Actinobacteria*, *Bifidobacterium*, *Streptococcaceae*, and *Muribaculaceae*, which were present at higher levels in the control and AEPE-M groups than in the CDAHFD group, were negatively correlated with prostaglandin H2, deoxycytidine, methylmalonic acid, indoleacrylic acid, L-ornithine, 5-hydroxytryptamine, 3-hydroxy-3-methylbutanoic acid, taurine, 2-(3,4-dimethoxyphenyl) ethanamine, and taurochenodeoxycholic acid. *Romboutsia*, *Peptostreptococcaceae*, *Desulfobacterota*, *Erysipelatoclostridiaceae*, *Erysipelatoclostridium*, *Faecalibaculum*, and *Lachnospiraceae*, which were present at higher levels in the CDAHFD group than in the control and CDAHFD groups, were negatively correlated with glutathione disulfide, N-lactoyl-phenylalanine, 3-indoleacrylic acid, and glucose 1-phosphate.

## 4 Discussion

A multitude of animal models are currently used to study NAFLD, including genetic leptin-deficient (ob/ob) or leptin-resistant (db/db) mouse and dietary MCD mouse models (Matsumoto et al., 2013). MCD model mice exhibit numerous pathological changes associated with NAFLD. It also leads to rapid weight loss and liver atrophy, which is inconsistent with the phenotype of human patients with NAFLD. In contrast, the CDAHFD model has been shown to simulate human NAFLD characteristics while overcoming the problems that arise when using the MCD model (Matsumoto et al., 2013). Therefore, CDAHFD can be used to establish a mouse model of rapid progressive liver fibrosis, which is more conducive to understanding the pathogenesis of NAFLD and facilitating the development of effective drugs or dietary supplements. In the present study, model mice incurred remarkable disorders in the levels of AST, ALT, HDL-C, LDL-C, TG and Hyp after CDAHFD feeding. The results of H&E staining, Oil Red O staining, and Masson’s trichrome staining showed that hepatocytes in the CDAHFD group displayed severe steatosis, obvious cell damage, and obvious deposition of collagen. AEPE treatment significantly improved liver steatosis and alleviated fibrosis.

The fecal microbiota transplantation (FMT) experiment confirmed that obesity and metabolic syndrome were improved by regulating the intestinal microbiota, and thus, it could be considered a new organ involved in the pathophysiology of NAFLD (Aron-Wisnewsky et al., 2020). Based on accumulating evidence, regulating intestinal flora using probiotics or prebiotics is a practical and effective method to prevent or treat gut microbiota-related diseases (Xue et al., 2017; Suk and Kim, 2019). As the intermediate phenotype between host and bacteria, changes in intestinal microbial metabolites play an essential role in understanding the effects of intestinal flora on diseases (Shi et al., 2022). Hence, treatments designed to regulate the intestinal microflora composed of intestinal microorganisms and microbial metabolites have become a research hot spot to prevent the development and deterioration of NAFLD.

In this study, we investigated the effect of the AEPE intervention on the intestinal flora of NAFLD mice. During the development of progressive hepatic fibrosis in NAFLD mice, AEPE reversed the changes in the diversity of the intestinal flora and adjusted the levels of components of the intestinal flora to restore them to normal levels. A clinical study showed that the intestinal microflora of patients with NAFLD is dominated by *Firmicutes* and *Bacteroidetes*, followed by *Proteobacteria* and *Actinobacteria*; however, *Proteobacteria* markedly increased in abundance, while the abundance of *Firmicutes* decreased as the disease progressed to advanced fibrosis (Loomba et al., 2017). Meanwhile, another report showed that the proportion of F/B in patients with NAFLD is increased (Li et al., 2021a). In this experiment, AEPE reversed the increase in the F/B ratio caused by CDAHFD. *Bifidobacterium* is a beneficial bacterium in the phylum *Actinobacteria*. Increased growth of *Bifidobacterium* contributes to the production of butyrate and other short-chain fatty acids (SCFAs) (Fang et al., 2021), which inhibit the production of proinflammatory factors and have the potential to contribute to the maintenance of host body weight and intestinal homeostasis, as well as improve the glucose and lipid metabolism (Bashiardes et al., 2016). *Bifidobacterium bifidum* and *Bifidobacterium adolescentis* inhibit hepatic inflammation and ameliorate NAFLD caused by a high-fat diet (HFD) by regulating intestinal microflora and increasing the content of propionic acid (Zhang et al., 2022). Meanwhile, transplantation with different strains of *Bifidobacterium* alleviates liver injury induced by various factors (He et al., 2022a; Zha et al., 2022). Ellagic acid, an active compound in PE, increases the relative abundance of *Bifidobacterium* in an alcoholic liver injury model (Zhao et al., 2021a). Compared with the control group, the levels of *Actinobacteria* and *Bifidobacterium* decreased significantly in the CDAHFD group but increased obviously after AEPE treatment. Moreover, a preliminary study has evaluated the effect of the PE extract on the intestinal flora in ob/ob mice (Brochot et al., 2019). In the experiment, the relative abundance of *Eubacterium* in the genus level of mice treated with PE extract was significantly increased, but other altered gut microbes were unidentified. In the present study, there was no significant difference in the relative abundance of *Eubacterium* among groups, and we speculate that this may be related to the difference in the induction factors of the model animals.

Additionally, the observation of clinical samples showed that the abundance of *Alistipes* in patients with NAFLD presenting liver fibrosis was lower than that in healthy patients (Rau et al., 2018). It was found in the HFD model and high-fat and high-fructose diet model mice that the relative abundance of *Alistipes* was significantly reduced compared with that in the control group (Wang et al., 2022; Yan et al., 2022). In addition, studies of mice with liver cancer have shown that *Alistipes* assists in inhibiting Th17 cells in the intestine and ultimately reduces Th17 cell recruitment to the liver, thereby reducing liver inflammation and improving liver fibrosis (Parker et al., 2020). At the same time, *Alistipes* also produces acetate and SCFAs with anti-inflammatory effects (Li et al., 2021b). Upon AEPE-M treatment, the relative abundance of *Alistipes* significantly increased compared with the CDAHFD-fed mice. Hence, we speculate that *Alistipes* may alleviate NAFLD by reducing intestinal inflammation and maintaining intestinal balance.

In addition, intervention with AEPE-M reversed the changes in other bacteria in CDAHFD mice. *Muribaculaceae*, a probiotic that produces SCFAs and o-glycans, maintains the dynamic balance of the intestinal mucosal barrier and assists in microbial colonization (Liang et al., 2021). In our experiment, compared with NAFLD mice, the relative abundance of *Muribaculaceae* in AEPE-M mice was obviously increased. Zhao et al. found that ellagic acid increases the relative abundance of *Muribaculaceae* in a mouse alcoholic liver injury model (Zhao et al., 2021a). Notably, gallic acid is an important marker component in AEPE. After FMT with feces from mice orally administered gallic acid, the relative abundance of *Muribaculaceae* in the gallic acid–FMT group was increased (He et al., 2022b). A clinical study showed that the abundance of the *Lachnospiraceae* family was significantly increased in fecal samples from patients with NAFLD compared with that in fecal samples from healthy subjects (Shen et al., 2017). In the present study, the highest relative abundance of *Lachnospiraceae* was observed in the CDAHFD group, consistent with that in a clinical report. Nevertheless, in our experiment, the relative abundance of *Faecalibaculum* in mice fed with CDAHFD was significantly increased compared with that in the control mice, and the trend was reversed in mice fed with AEPE-M. However, a study showed that in animals with NAFLD induced by an HFD, the relative abundance of *Faecalibaculum* in HFD-fed mice was significantly reduced compared with that in the control group (Hu et al., 2021). We assume that the explanation for this phenomenon is the induction of NAFLD by different factors. Members of the *Erysipelotrichaceae* family are closely related to clinical indicators of impaired glucose and lipid metabolism and are important targets of metabolic diseases (Zhao et al., 2021b). The reduction in *Erysipelotrichaceae* may be beneficial for lipid metabolism. Taken together, AEPE-M can be used to reduce the degree of steatosis and fibrosis in NAFLD mice by modulating bacteria at multiple levels.

Changes in intestinal flora must be accompanied by alterations in different metabolites, such as bile acids, amino acids, and SCFAs. The metabolites of the intestinal flora have become an important factor regulating the pathological process of NAFLD. Therefore, this study examined the effect of AEPE on fecal metabolites. Fourteen differential metabolites were identified, including 5-hydroxytryptamine, glucose 1-phosphate, and L-ornithine. Based on the result of the correlation analysis, the levels of the aforementioned metabolites, namely, glutathione disulfide and N-lactoyl-phenylalanine, were positively correlated with the abundance of *Actinobacteria*, *Bifidobacterium*, *Streptococcaceae*, and *Muribaculaceae*.

A study reported the dysregulation of bile acid metabolism in patients with NAFLD, including elevated levels of primary conjugated bile acids (BAs), decreased levels of specific secondary Bas, and alteration of excreted BAs (Masoodi et al., 2021). Primary BAs are conjugated to glycine or taurine before being secreted into the bile for storage. The conjugated primary BAs are then released into the small intestine to facilitate lipid absorption and conversion to secondary bile acids by intestinal microorganisms. Secondary BAs are reabsorbed into the liver via the liver–gut circulation to inhibit bile acid synthesis. The continuous intrahepatic circulation of BAs ensures the steady state of BAs and the physiological function of the hepatic–intestinal axis (Li et al., 2020). Based on the results from this experiment, we found significantly higher contents of taurine and taurochenodeoxycholic acid in the feces of mice fed the CDAHFD than in those of mice fed the control diet, while these changes were reversed after AEPE consumption. We suggested that AEPE modifies the metabolic disorders caused by CDAHFD by regulating primary bile acid biosynthesis and taurine and hypotaurine metabolism.

Tryptophan metabolism is associated with a variety of diseases, including NAFLD, metabolic syndrome, obesity, and irritable bowel syndrome (Pyun et al., 2021). In the present study, several differential metabolites were related to the tryptophan metabolism pathway, such as 5-hydroxytryptamine (5-HT), indoleacrylic acid, and 3-indoleacrylic acid. Over 90% of peripheral 5-HT is produced in the gut (Agus et al., 2018). Actually, the enteric microorganism has been proved to be a primary contributor to the production of 5-HT in the gut (Ni et al., 2020). According to previous studies, a 5-HT antagonist effectively improves fatty and fibrotic changes in a mouse NAFLD model induced by CDAHFD (Ko et al., 2021). Interestingly, our findings manifested that AEPE significantly reduced the increase in the fecal 5-HT content observed in CDAHFD mice, suggesting that AEPE may ameliorate hepatic steatosis and fibrosis by reducing 5-HT production in the gut. Indoleacrylic acid is a compound from a group of molecules known as indoles. Intestinal microorganisms catabolize tryptophan to produce indole derivatives, which are then absorbed and converted into indoleacrylic acid. Indole and its derivatives are beneficial in the treatment of liver diseases; for example, oral indole administration inhibits the NF-*κ*B pathway and reduces LPS-induced inflammation in the liver (Beaumont et al., 2018). Yu et al. suggested significant differences in indoleacrylic acid levels between rats with hepatic fibrosis and normal rats, although its biological role in liver pathology remains unclear (Yu et al., 2019). 3-Indoleacrylic acid, a tryptophan metabolite (Roager and Licht, 2018), activates the aryl hydrocarbon receptor (AHR) and thus maintains the integrity of the intestinal barrier (Li et al., 2021c). Increased intestinal permeability exacerbates the development of NAFLD (Portincasa et al., 2022); therefore, AEPE may improve NAFLD by regulating the balance of intestinal microecology and decreasing intestinal permeability. The correlation analysis revealed that 3-indoleacrylic acid levels were positively correlated with the abundance of *Muribaculaceae* and negatively correlated with the abundance of *Romboutsia*, *Peptostreptococcaceae*, and *Faecalibaculum*. Consequently, we presumed that the effect of AEPE on tryptophan metabolism may be related to its regulation of the abundance of *Muribaculaceae*, *Romboutsia*, *Peptostreptococcaceae*, and *Faecalibaculum*.

Glucose-1-phosphate (G-1-P) is the product of the reaction in which glycogen phosphorylase cleaves molecules of glucose from the larger glycogen structure. Our results showed that decreased G-1-P levels in NAFLD mice were inversely regulated following AEPE treatment, which may be mediated by *Muribaculaceae*, *Romboutsia*, *Peptostreptococcaceae,* and *Faecalibaculum*. In the present study, G-1-P is a common key metabolite involved in multiple metabolic pathways, including starch and sucrose metabolism, amino sugar and nucleotide sugar metabolism, and galactose metabolism. Among them, the dysfunction of amino sugar and nucleotide sugar metabolism has been reported in individuals with various metabolic diseases, such as dyslipidemia, type 2 diabetes, and NAFLD (Huang et al., 2020; Li et al., 2022b). However, evidence to clarify the relationship between the metabolic pathway and NAFLD in previous studies is insufficient. The potential mechanism by which AEPE ameliorates NAFLD by regulating multiple metabolic pathways mediated by GP is worthy of in-depth investigation. Methylmalonic acid (MMA), a malonic acid derivative, is a crucial intermediate in lipid and protein metabolism. Abnormal accumulation of MMA disrupts normal glucose and glutamic acid metabolism in the rat liver (Toyoshima et al., 1996). Another study proposed MMA as a biomarker reflecting the degree of hepatic mitochondrial fatty acid oxidation in rats (Bjune et al., 2021). AEPE treatment significantly decreased the MMA content in the feces of NAFLD mice. Therefore, our results also provide clues to further explore the role of MMA in the pathogenesis of NAFLD.

Moreover, L-ornithine is involved in glutathione metabolism, arginine biosynthesis, and arginine and proline metabolism. Dysfunctional glutathione metabolism may lead to excessive reactive oxygen species production, aggravate lipid peroxidation and oxidative stress, and exacerbate liver injury. In addition, glutathione metabolism has been proven to be one of the main mechanisms affecting hyperlipidemia (Xiao-Rong et al., 2021). Thus, we hypothesized that AEPE may ameliorate NAFLD by modulating L-ornithine-mediated glutathione metabolism, reducing the accumulation of lipids in the liver, and increasing the antioxidant capacity of the body.

In summary, our studies have identified various effects of AEPE on ameliorating NAFLD, including reducing hepatic steatosis, improving dyslipidemia, and reducing fibrosis. The mechanism of AEPE in the treatment of NAFLD is associated with reshaping the intestinal microecology.

## Data Availability

The datasets presented in this study can be found in online repositories. The names of the repository/repositories and accession number(s) can be found at: NCBI BioProject—PRJNA821399.
